# Alcohol, aging, and the gut microbiome: Intersections of immunity, barrier dysfunction, and disease

**DOI:** 10.1016/j.alcohol.2025.07.001

**Published:** 2025-07-09

**Authors:** Esther Melamed, Wiramon Rungratanawanich, Suthat Liangpunsakul, Katherine A. Maki, Rebecca L. McCullough, Cristina Llorente

**Affiliations:** a Department of Neurology, The University of Texas at Austin, Dell Medical School, Austin, TX, USA; b Section of Molecular Pharmacology and Toxicology, National Institute on Alcohol Abuse and Alcoholism, Bethesda, MD, USA; c Division of Gastroenterology and Hepatology, Department of Internal Medicine, Indiana University School of Medicine, Indianapolis, IN, USA; d Department of Biochemistry and Molecular Biology, Indiana University School of Medicine, Indianapolis, IN, USA; e Roudebush Veterans Administration Medical Center, Indianapolis, IN, USA; f Biobehavioral and Integrated MetagenOMics (BIOM) Unit, Translational Biobehavioral and Health Disparities Branch, National Institutes of Health Clinical Center, Bethesda, MD, USA; g Skaggs School of Pharmacy and Pharmaceutical Sciences, University of Colorado Anschutz Medical Campus, Aurora, CO, USA; h Division of Gastroenterology and Hepatology, Department of Medicine, University of California San Diego, La Jolla, CA, USA

**Keywords:** Alcohol, Microbiome, Gut-liver axis, Gut-brain axis, Neuroinflammation

## Abstract

Alcohol consumption exerts complex, dose- and context-dependent effects on human health, particularly by influencing the gut microbiome, intestinal barrier integrity, immune regulation, and aging processes. Genetic variation and advancing age are two major, and often interacting, factors that modify the risk of alcohol-related diseases. Among genetic factors, the prevalent aldehyde dehydrogenase 2 polymorphism (ALDH2*2) compromises acetaldehyde clearance, driving toxic metabolite accumulation, oxidative stress, and increased intestinal permeability that disrupts gut microbial communities, even at low levels of alcohol consumption. Heavy and chronic alcohol use further disrupts gut microbial communities, erodes mucosal integrity, and drives systemic inflammation, contributing to alcohol-associated liver disease (ALD), neuroinflammation, and multi-organ injury. Aging independently worsens these effects by promoting chronic low-grade inflammation and impaired immune responses, heightening susceptibility to alcohol-induced pathology. In specific contexts, such as certain autoimmune diseases, low to moderate alcohol intake may exert immunomodulatory effects and influence the gut microbiome, potentially contributing to reduced inflammation and alterations in microbial composition. This review synthesizes current mechanistic insights into how alcohol, host genetics, the gut microbiome, immune regulatory pathways, and aging intersect to influence disease risk. As global populations age and the burden of alcohol-related health issues rises, there is an urgent need for integrated, systems-level approaches. Future research should prioritize precision-based, gut-targeted strategies aimed at restoring microbial balance, maintaining intestinal barrier integrity, and mitigating alcohol-related harm across the lifespan.

## 1. Introduction

Alcohol use exerts complex, dose-dependent effects on human health, spanning from detrimental consequences in the setting of heavy or chronic consumption (Room et al., 2005). At the center of these alcohol-mediated effects lies the dynamic interplay between alcohol metabolism, host genetic factors, gut barrier integrity, and the composition and function of the intestinal microbiota (Carbia et al., 2023; Khan & Chang, 2023; McMahan et al., 2023; Raya Tonetti et al., 2024). Notably, the gut-liver, gut-lung and gut-brain axes have emerged as critical mechanistic links connecting alcohol-related alterations in the gut and lung environments to systemic inflammation, liver and lung injury, neuroinflammation, and immune dysregulation (Gao et al., 2024; Raya Tonetti et al., 2024; Yan et al., 2023). Beyond pathological states, a growing body of literature has begun to explore the immunomodulatory effects of alcohol consumption, particularly its influence on autoimmune disease risk and progression (Terracina et al., 2025a). The health effects of alcohol are further complicated by aging; advancing age is characterized by chronic low-grade inflammation, increased intestinal permeability, and microbiome alterations, all of which are independently worsened by alcohol exposure (McMahan et al., 2021, 2023). In older adults, the combination of age-related intestinal barrier dysfunction, microbial dysbiosis, and heightened inflammatory responses synergizes with alcohol-induced injury, increasing susceptibility to multi-organ damage, neuroinflammation, and impaired cognitive function (McMahan et al., 2021, 2023). This review synthesizes current knowledge regarding the interconnected roles of alcohol metabolism, gut barrier integrity, microbiome composition, immune regulation, lung and liver injury, and aging in shaping health outcomes related to alcohol use.

### 1.1. Ethanol oxidation, acetaldehyde toxicity, and the gut-liver-brain axis in ALDH2 deficiency

Upon ingestion, alcohol is absorbed by the gastrointestinal (GI) tract through diffusion into the blood vessels and distributed throughout the body via the bloodstream. The majority of ethanol undergoes both oxidative and non-oxidative metabolism, while the remainder is eliminated through breath, sweat, and urine (Cederbaum, 2012; Holford, 1987; Israelsen et al., 2024). Alcohol metabolism primarily occurs via the oxidative pathway in the liver and involves a series of enzymatic reactions. Ethanol is first converted to acetaldehyde by three key enzymes: alcohol dehydrogenase (ADH), catalase, and cytochrome P450–2E1 (CYP2E1). Cytosolic ADHs, especially class I ADHs, are primary enzymes responsible for alcohol oxidation, using nicotinamide adenine dinucleotide (NAD^+^) as a cofactor. Another enzyme, catalase, located in cellular structures known as peroxisomes, can also metabolize alcohol in the presence of hydrogen peroxide (H_2_O_2_) (Le Dar et al., 2019; Ramchandani et al., 2001). However, catalase does not have a large role in alcohol oxidation due to the low availability of H_2_O_2_ in the liver (Lieber, 2000). A third pathway involves CYP2E1, which becomes more active when subjects binge alcohol. CYP2E1 is present within the microsomal ethanol-oxidizing system (MEOS). CYP2E1 uses nicotinamide adenine dinucleotide phosphate (NADPH) as a cofactor, resulting in the generation of oxygen species (ROS) as metabolic byproducts. These ROS contribute to oxidative stress and play a significant role in liver injury (Rungratanawanich et al., 2021, 2023a; Song et al., 2019). Following initial metabolism, acetaldehyde is produced as a toxic intermediate that requires rapid conversion to less toxic compounds. Acetaldehyde is oxidized to acetate by aldehyde dehydrogenase 2 (ALDH2), a mitochondrial enzyme with high affinity for acetaldehyde to facilitate clearance (LeFort et al., 2024; Zakhari, 2006). Finally, acetate is further catabolized to carbon dioxide (CO_2_) and water (H_2_O), which are then cleared from the body. In addition to its role in alcohol metabolism, ALDH2 removes other harmful compounds like lipid aldehydes. This combined cellular protection and alcohol metabolism roles of ALDH2 highlight this enzyme as important in safeguarding against disease processes associated with alcohol use disorder (AUD) (Rungratanawanich et al., 2021; Song et al., 2015).

While the brain contains the same alcohol-metabolizing enzymes found in the liver (e.g., ADH, catalase, CYP2E1, and ALDH2), these enzymes play very different roles in brain alcohol metabolism. Unlike in the liver, ADH plays little to no role in brain alcohol metabolism. Instead, catalase accounts for 60 % of ethanol conversion to acetaldehyde under normal conditions (Zimatkin et al., 2006). However, this percentage may be overestimated because catalase inhibitors used in research can also inhibit CYP2E1 at high ethanol concentrations, which may lead to an overinflation of the role of catalase in brain ethanol metabolism (Arizzi-LaFrance et al., 2006; Zimatkin & Lindros, 1996). CYP2E1 is widely expressed in various brain regions, including the prefrontal cortex, hippocampus, amygdala, brainstem, and cerebellum, and is inducible by alcohol exposure, making it a likely key contributor to brain ethanol metabolism, particularly following chronic or binge drinking (Rungratanawanich et al., 2021; Upadhya et al., 2000; Yadav et al., 2006). Additionally, ALDH2 is expressed in multiple brain areas, such as the frontal cortex, midbrain, hippocampus, and temporal cortex (Alnouti & Klaassen, 2008; Picklo et al., 2001; Zakhari, 2006), where it plays a crucial role in detoxifying acetaldehyde to acetate, which is subsequently catabolized to carbon dioxide and water (Vadigepalli & Hoek, 2018).

More than half a billion people worldwide have reduced ALDH2 activity due to a dominant-negative mutation in the ALDH2 gene (ALDH2*2) (Chen et al., 2020; Gill et al., 1999; Wall et al., 2000). Individuals with ALDH2 deficiency commonly experience physiological symptoms such as facial flushing, tachycardia, and palpitations after alcohol consumption, largely attributed to the accumulation of toxic compounds, including acetaldehyde, reactive lipid aldehydes, and acetaldehyde adducts (Brooks et al., 2009; Yokoyama et al., 2003). This genetic deficiency increases susceptibility to alcohol-induced multi-organ damage involving the gut, liver, and brain, elevates the risk for developing certain cancers, particularly of the oral cavity and GI tract (Chen et al., 2020; Gill et al., 1999; Wall et al., 2000), and heightens vulnerability to late-onset neurodegenerative diseases, including Alzheimer’s and Parkinson’s diseases (D’Souza et al., 2015; Deza-Ponzio et al., 2018; Xia et al., 2020). In experimental models, *Aldh2* gene deletion or knockout (KO) mice have been developed to study the functional role of ALDH2. These mice exhibit minimal to absent ALDH2 activity—mirroring human deficiency—and demonstrate significantly higher acetaldehyde accumulation in the blood, liver, and brain following alcohol exposure (Isse et al., 2005; Rungratanawanich, Lin, et al., 2023). Like humans with ALDH2 deficiency, *Aldh2*-KO mice display increased sensitivity to alcohol-induced multi-organ injury, DNA damage, inflammatory liver diseases, neurodegeneration, cognitive impairment, cancer development, and reduced lifespan (Jin et al., 2021; Matsumoto et al., 2014; Rungratanawanich, Lin, et al., 2023; Yu et al., 2009; Yukawa et al., 2014). The parallels between ALDH2-deficient individuals and *Aldh2*-KO mice underscore the protective role of ALDH2 and reflect a conserved biological phenomenon across species.

In the GI tract, alcohol consumption is commonly associated with gut microbiota dysbiosis, small intestinal bacterial overgrowth, and increased intestinal permeability, collectively contributing to the translocation of pathogen-associated molecular pattern (PAMP) molecules, such as endotoxin (lipopolysaccharide or LPS, produced by gram-negative bacteria), into the bloodstream (Keshavarzian et al., 2009; Lowe et al., 2020). Elevated LPS levels within the gut can initiate local inflammation by activating the immune system in the lamina propria, while increased circulating LPS, or endotoxemia, can trigger systemic inflammation, leading to multi-organ injury, including the liver and brain (Keirns et al., 2024). These pathological features, microbiota dysbiosis, gut leakiness, and endotoxemia, are consistently observed in both preclinical models and individuals with AUD (Leclercq et al., 2014; Maki et al., 2024), including those with alcohol-associated liver disease (ALD) and alcohol-related brain damage (ABD) (Carbia et al., 2023; Kalyan et al., 2022; Leclercq et al., 2014; Stä et al., 2018; Xie et al., 2023).

Emerging evidence highlights the interplay between intestinal microbiome dysbiosis and alcohol metabolism. Under physiological conditions, the absence of ALDH2 has been associated with reductions in beneficial bacterial populations, including Verrucomicrobia (which contains *Akkermansia* (*A*.) *muciniphila*), *Actinobacteria*, and *Lactobacillus*, alongside increases in opportunistic gram-negative bacteria such as *Deferribacteres* and *Proteobacteria* species (Li et al., 2023; Rungratanawanich, Lin, et al., 2023; Yang, Chen, et al., 2021). These microbial imbalances and the overgrowth of gram-negative bacteria may represent a hidden mechanism contributing to the heightened severity of ALDH2 deficiency-mediated multi-organ injury. Supporting this, experimental studies in *Aldh2*-KO mice have demonstrated significant increases in serum LPS levels and intestinal permeability following even a single, low-dose alcohol exposure—effects not observed in wild-type (WT) controls (Rungratanawanich, Lin, et al., 2023). ALDH2 depletion also elevates intestinal oxidative and nitrative stress, promoting post-translational protein modifications (e.g., protein nitration) that drive the degradation of tight junction and adherens junction proteins via ubiquitin-dependent proteolysis, induce enterocyte apoptosis, and ultimately result in gut leakiness and endotoxemia (Rungratanawanich, Lin, et al., 2023). The subsequent rise in circulating endotoxin, originating from gut dysbiosis, exacerbates systemic inflammation and contributes to downstream organ damage, including in the liver and brain. Notably, *Aldh2*-KO mice display greater severity in alcohol-induced intestinal permeability, inflammatory liver injury, and neurodegeneration compared to WT mice exposed to equivalent ethanol doses (Ray et al., 2024; Rungratanawanich, Lin, et al., 2023). These pathological changes are characterized by increased intestinal inflammation, enhanced gut permeability, and elevated serum LPS, accompanied by exacerbated hepatic inflammation, apoptosis, and neuronal oxidative damage (Ray et al., 2024; Rungratanawanich, Lin, et al., 2023). In addition to alterations in the intestinal microbiome, disruptions in bacterial metabolites, such as decreased short-chain fatty acid (SCFA) levels, may also contribute to the increased susceptibility to alcohol-induced multi-organ injury in the context of ALDH2 deficiency, although further investigation is needed.

### 1.2. Gut barrier integrity, mucin Glycosylation, and goblet cell dynamics in the pathogenesis of ALD

Chronic alcohol consumption disrupts the gut-liver axis, resulting in intestinal dysbiosis, increased gut permeability, and bacterial translocation to the liver. These alterations trigger immune activation, leading to hepatic inflammation and progressive liver injury (Bode et al., 1987; Hartmann et al., 2012; Llorente, 2024; Llorente & Schnabl, 2015; Yan et al., 2011). Recent research has identified the gut microbiome as a promising therapeutic target in ALD. For instance, microbiome alterations that promote intestinal overgrowth of *Enterococcus* have been linked to an increased risk of ALD (Llorente et al., 2017). Bacteriophage therapy directed against virulent *Enterococcus faecalis* strains expressing a pore-forming toxin, cytolysin, has been shown to reduce liver injury in experimental models (Duan et al., 2019), while fecal microbiota transplantation (FMT) has demonstrated encouraging outcomes in patients with severe alcohol-associated hepatitis (Table 1) (Philips et al., 2022; Wolstenholme et al., 2024).

A key player in maintaining gut health and regulating mucosal immune responses is the goblet cell. These specialized epithelial cells are distributed throughout the GI tract, with a higher density in the colon, and play a critical role in sustaining the intestinal barrier by continuously secreting mucus (Bergstrom et al., 2010; Tonetti et al., 2024). This mucus layer, primarily composed of mucin glycoproteins, serves as a physical and biochemical barrier that protects against pathogenic invasion while supporting a healthy microbiome. Among these mucins, Mucin-2 (MUC2) is the predominant form in the gut, and its production has been implicated in the pathogenesis of alcohol-induced liver injury. Interestingly, patients with AUD exhibit increased duodenal mucus thickness (Hartmann et al., 2013), and studies in Muc2-deficient mice reveal reduced ethanol-induced liver injury and bacterial overgrowth, highlighting the complex and context-dependent role of mucins in ALD (Hartmann et al., 2013). Goblet cells also exhibit functional diversity based on their subtypes. Of particular interest are sentinel goblet cells, located at the top of distal colonic crypts, which actively participate in microbial sensing through endocytosis of bacterial products. Upon detecting a threshold level of microbial signals, sentinel goblet cells initiate a cascade that prompts neighboring goblet cells to rapidly secrete mucus, reinforcing the intestinal barrier and preventing microbial translocation (Birchenough et al., 2016). In the context of liver disease, goblet cell dynamics are altered across the spectrum of cirrhosis. Compensated cirrhosis is associated with increased goblet cell abundance, whereas advanced decompensated cirrhosis shows a marked reduction in goblet cell numbers. Gene expression analyses from these patients reveal upregulation of pro-inflammatory cytokines, diminished expression of goblet cell differentiation markers, and paradoxically, increased MUC2 production in decompensated states (Jiang et al., 2024). These findings underscore the complex, stage-dependent interplay between goblet cell biology, mucin production, and gut-liver axis dysfunction in chronic liver disease.

Mucin glycoproteins are heavily decorated with complex glycans, including fucosylated glycans, which play an essential role in maintaining intestinal homeostasis and protection from alcohol-associated damage. Notably, patients with AUD exhibit a reduction in intestinal α1–2-fucosylation—a specific biochemical modification where fucose residues are attached to intestinal glycans via α1–2 linkages (Zhou et al., 2020). This modification is catalyzed by the enzyme alpha-1, 2-L-fucosyltransferase encoded by the *Fut2* gene. In preclinical models, *Fut2*-deficient mice lack the ability to perform α1–2-fucosylation and display increased susceptibility to alcohol-induced liver damage, steatosis, and inflammation (Zhou et al., 2020). Importantly, α1–2-fucosylation contributes to shaping the intestinal microbiome by reducing the intestinal burden of pathogenic *Enterococcus faecalis*, particularly strains that produce the hepatotoxin cytolysin in the context of alcohol exposure. These findings suggest that interventions aimed at enhancing intestinal fucosylation may offer protection against microbiome-driven liver injury in AUD. In this regard, 2′-fucosyllactose, a naturally occurring fucosylated oligosaccharide, has emerged as a potential therapeutic candidate for mitigating ALD (Table 1) (Zhou et al., 2020). Alcohol misuse also leads to a depletion of *A. muciniphilia*, a mucin-degrading bacterium that plays a key role in the maintenance of GI homeostasis. For example, *A. muciniphilia* contributes to gut barrier integrity and immune regulation through consumption-associated mucin renewal and regulation of anti-inflammatory cytokine release. The loss of this species of bacteria disrupts the balance of microbial metabolites, weakens the intestinal barrier, and impairs mucin secretion, resulting in increased gut permeability (Grander et al., 2018; Sparfel et al., 2024). The compromised barrier promotes microbial translocation and systemic exposure to pro-inflammatory microbial products, triggering chronic inflammation and cytokine release, which further amplifies intestinal and systemic immune responses.

Goblet cells have long been recognized for their essential role in secreting mucins and maintaining the intestinal barrier. In addition to these functions, goblet cells are crucial in regulating gut immune responses (Knoop et al., 2020; Kulkarni et al., 2020). They achieve this by forming goblet cell-associated antigen passages (GAPs), which facilitate the transport and regulated sampling of luminal antigens to innate immune cells located in the lamina propria (McDole et al., 2012). GAP formation is highly regulated and is most prominent in the small intestine, while it is suppressed in the proximal colon (Kulkarni et al., 2020). This process is modulated by acetylcholine through muscarinic acetylcholine receptor 4 (mAChR4) signaling (Knoop et al., 2015; McDole et al., 2012). While chronic alcohol consumption increases the number of mucin-producing goblet cells, it also impairs GAP formation in the small intestine by downregulating the expression of *Chrm4* mRNA and its encoded protein, mAChR4. This downregulation reduces the number and function of tolerogenic dendritic cells, leading to weakened anti-microbial immune responses. The weakened immune function decreases interleukin (IL)-22 signaling in group 3 innate lymphoid cells (ILC3) and reduces production of regenerating antimicrobial peptides (Reg3), ultimately allowing more bacteria to cross into the bloodstream and trigger liver inflammation (Llorente et al., 2021).

Indeed, growing evidence implicates the gut-liver axis as a central driver in ALD pathogenesis, with goblet cells and mucin glycoproteins playing pivotal roles in preserving intestinal barrier integrity and regulating immune responses. Disruptions in mucin production, fucosylation, and GAP formation contribute to increased bacterial translocation, persistent inflammation, and progressive liver injury. Emerging therapeutic strategies targeting these pathways, including bacteriophage therapy, FMT, and 2′-fucosyllactose supplementation, show considerable promise in preclinical and early clinical investigations. Future research should prioritize translating these mechanistic insights into effective microbiome-based interventions aimed at restoring gut homeostasis and attenuating the progression of ALD. By addressing these complex gut-liver interactions, these novel strategies hold the potential to improve clinical outcomes and reduce the substantial global burden of ALD.

### 1.3. Alcohol-mediated gut dysbiosis, immune disruption, endotoxemia, and pneumonia risk in the gut-lung axis

The influence of the gut microbiome on human health extends beyond the well-characterized gut-liver-brain axis. Emerging evidence supports the existence of a gut-lung axis, particularly relevant in the context of alcohol-induced acute lung injury. Chronic and binge ethanol exposure disrupts the gut microbiome by promoting bacterial overgrowth and dysbiosis, resulting in alterations in microbial metabolism, dysregulation of mucosal immunity in the small intestine, and impairment of the intestinal epithelial barrier (Bruellman & Llorente, 2021). These alterations facilitate the translocation of microbial metabolites and endotoxins from the intestinal lumen into the circulatory and lymphatic systems, ultimately reaching the lungs (Santilli, Shapiro, et al., 2024). Furthermore, ethanol exposure perturbs immune responses across the gut, blood, and lungs. Ethanol suppresses CD3^+^CD8a^+^ T cells in the colon and CD11c^+^CD8a^+^ dendritic cells in the mesenteric lymph nodes, while increasing the levels of Ly6G^+^CD11b^+^ neutrophils in the circulation. Within the lungs, elevated populations of Ly6G^+^CD11b^+^ neutrophils and CD11c^+^CD64^+^ macrophages are observed, accompanied by increased expression of oxidative stress markers, including lipocalin-2 and myeloperoxidase (Santilli, Han, et al., 2024). The consequences of gut dysbiosis-induced endotoxemia in the gut/liver/lung axis have been previously reviewed (Massey et al., 2015). Briefly, the elevated systemic endotoxin levels can promote pulmonary vasoconstriction, leukocyte sequestration, oxidation of mitochondrial thioredoxin-2, and mitochondrial dysfunction, highlighting the implication of oxidative stress. Additionally, there is evidence to suggest that hepatic-derived mediators, such as tumor necrosis factor-alpha (TNF-α), may also play a role in the pathogenesis of alcohol-induced acute lung injury (Massey et al., 2015), although additional studies are necessary to confirm these findings. Together, these processes contribute to alcohol-related lung dysfunction and heightened susceptibility to lung diseases. Mice recolonized with a microbiota from alcohol-fed mice showed higher respiratory *Klebsiella pneumoniae* burden, increased pulmonary inflammatory cytokines, and reduced CD4^+^ and CD8^+^ T-cells in the lungs (Samuelson et al., 2017). Similarly, administration of IgA-coated bacteria from ethanol-fed mice impaired host defense against *Streptococcus pneunomiae* (Gu et al., 2021). Furthermore, the risk of pneumonia is increased by alcohol-induced reductions in peripheral natural killer (NK) cells and their cytolytic activity (Villageliu et al., 2024). Numerous prospective and retrospective studies consistently show that AUD patients face an elevated risk of contracting pneumonia (de Roux et al., 2006; Gupta et al., 2019; Simou et al., 2018; Zhang et al., 2008). These findings highlight the critical interplay between the gut and the lung in the setting of alcohol exposure and emphasize the need to better understand the immune dysregulation that underlies alcohol-related respiratory complications.

### 1.4. Moderate alcohol consumption, autoimmunity, and the gut microbiome: mechanisms and clinical considerations

Excessive alcohol intake is well-established as a cause of liver damage, cardiovascular disease, increased susceptibility to infections, cancers, and neurodegeneration (Haber, 2025). While moderate alcohol consumption, defined by the National Institute on Alcohol Abuse and Alcoholism as up to one drink per day for women and up to two drinks per day for men (resulting in a blood alcohol concentration of <0.08 g/dL) (National Institute on Alcohol Abuse and Alcoholism), has been associated in some studies with certain anti-inflammatory cytokine responses in autoimmune diseases (Caslin et al., 2021; Terracina et al., 2025b), these observations remain under investigation. The mechanisms proposed to explain these associations include modulation of immune pathways and interactions with the gut microbiome.

Autoimmune diseases affect approximately 5–8% of the global population, representing a substantial and growing health burden worldwide (Fugger et al., 2020). These disorders are characterized by inappropriate inflammatory immune responses targeting self-antigens (Murphy et al., 2022). Epidemiological studies suggest a U-shaped, dose-dependent relationship between alcohol consumption and autoimmune disease risk and severity, where moderate consumption is associated with reduced risk compared to both abstinence and heavy drinking (Caslin et al., 2021). For example, in multiple sclerosis, moderate alcohol intake has been linked to lower disease risk and potentially slower progression (Andersen et al., 2019; Diaz-Cruz et al., 2017; Hedström et al., 2014; Malekifar et al., 2023). Similar associations have been observed in rheumatoid arthritis (RA), systemic lupus erythematosus, autoimmune diabetes, autoimmune thyroid disease, allergic rhinitis, and primary biliary cholangitis, with moderate alcohol consumption associated with reduced inflammatory markers and disease severity (Caslin et al., 2021; Terracina et al., 2025a). Additionally, animal studies have demonstrated moderate alcohol intake in models of experimental autoimmune encephalomyelitis (EAE), a widely used model for multiple sclerosis, was associated with greater disease remission and decreased microglial density, as well as in models of RA (Azizov et al., 2020; Caslin et al., 2019).

Mechanistically, moderate alcohol consumption may modulate immune function through several pathways. It has been shown to reduce the production of pro-inflammatory cytokines, including TNF-α, IL-1β, IL-6, TNF receptor 2, IL-21, and IL-17A, while promoting anti-inflammatory cytokines such as IL-10 (Azizov et al., 2020; Lu et al., 2010; Mandrekar et al., 2006; Romeo et al., 2007). Moderate alcohol intake has also been found to inhibit nuclear factor kappa B (NF-κB) activation, a central transcription factor in inflammatory signaling (Jonsson et al., 2007). Furthermore, moderate alcohol consumption has been associated with reductions in neutrophils, monocytes, plasma B cells, and IgG levels (Azizov et al., 2020), alongside influences on T cell differentiation, favoring the induction of regulatory T cells (Messaoudi et al., 2013) and suppressing T follicular helper cells (Azizov et al., 2020). Additionally, moderate alcohol may suppress dendritic cell activation and maturation, leading to reduced antigen presentation and T cell activation (Mandrekar et al., 2004; Thompson et al., 2016). Collectively, these immune-modulating effects may create an immunological environment that attenuates inflammatory autoimmune processes.

Beyond immunological effects, moderate alcohol consumption may influence autoimmune disease pathways via modulation of the gut microbiome. The gut microbiome, a vast community composed of trillions of bacteria, fungi, and protozoa, has been increasingly recognized as a key environmental factor in the initiation and progression of autoimmune diseases (Gonzá et al., 2020). Moderate alcohol intake has been shown to alter gut microbial composition by increasing *Bifido-bacterium* and *A*. *muciniphila*, while enhancing overall microbial diversity (Caslin et al., 2019; Queipo-Ortuñ et al., 2012). It may also contribute to the production of gut-derived microbial anti-inflammatory metabolites, particularly SCFAs such as acetate, propionate, and butyrate (Gonzá et al., 2020). These SCFAs can either be produced by gut bacteria (e.g., *Akkermansia*) or directly metabolized from alcohol, as acetate is an end-product of alcohol metabolism. Additionally, moderate alcohol intake influences the production of polyunsaturated fatty acids (PUFAs) (di Giuseppe et al., 2009), which have been shown to reduce reactive oxygen species and exert anti-inflammatory effects in autoimmune disease contexts. Both SCFAs and PUFAs can cross the gut barrier and mitigate inflammation in autoimmune diseases (Parada Venegas et al., 2019), providing a mechanistic link between moderate alcohol consumption, the gut microbiome, and immune regulation.

While these contextual findings are of interest, it is important to consider the comorbid conditions commonly observed in patients with autoimmune diseases, which may be adversely affected by alcohol consumption. These include metabolic-associated steatotic liver disease (Jophlin et al., 2024), metabolic syndrome (Åberg et al., 2020), and mental health disorders (Li et al., 2020). Moreover, even moderate alcohol consumption can exacerbate symptoms such as sleep disturbances and fatigue, which are frequently experienced by individuals with autoimmune diseases (Terracina et al., 2025b). Additionally, several immunomodulatory drugs used to manage autoimmune conditions may interact with alcohol metabolism or potentiate alcohol-related adverse effects (White et al., 2022; Yang et al., 2022). Defining safe consumption limits is further complicated by varying individual vulnerabilities to alcohol dependence.

Current research limitations on moderate alcohol include inconsistent definitions of “moderate” alcohol consumption across studies, potential confounding variables such as lifestyle factors, predominantly observational study designs that cannot establish causation, and insufficient animal studies that preclude a mechanistic understanding of how alcohol modulates autoimmune responses at the molecular level. Nevertheless, the growing recognition of the complex interplay between moderate alcohol consumption, the gut microbiome, and autoimmunity opens promising avenues for future investigation. One important question is to what extent moderate drinkers may be more likely to engage in health-promoting behaviors, such as regular physical activity, a balanced diet, and abstinence from smoking, termed the “healthy lifestyle effect,” that may independently improve health outcomes (Naimi et al., 2017; Stockwell et al., 2016). Further research is needed, including prospective human clinical studies examining the effects of moderate alcohol consumption on specific autoimmune diseases with comprehensive immune and microbiome profiling to better define the beneficial and detrimental effects of alcohol in autoimmune diseases at different doses. Preclinical studies in animal models of specific autoimmune disease should investigate distinct mechanistic effects of moderate alcohol intake, comparing the effects of different alcoholic beverage types and elucidating the mechanistic underpinnings of moderate alcohol relative to high-dose alcohol in autoimmune diseases, relative to other chronic diseases. Additionally, the development of microbiome-targeted interventions that replicate the contextual immunomodulatory effects of moderate alcohol consumption without alcohol’s use and associated risks represents a compelling therapeutic strategy. It will also be essential to explore the synergistic and potentially detrimental interactions between moderate alcohol intake and common autoimmune disease treatments, alongside patient-centered research focusing on quality of life, symptom burden, and moderate alcohol consumption patterns, as well as the interactions between moderate alcohol and healthy lifestyle factors.

### 1.5. Alcohol, aging, and the gut: intersecting pathways of barrier dysfunction, inflamm-aging, and disease risk

While alcohol misuse occurs across the lifespan, its specific health effects in advancing age (65 years or older) remain poorly defined. As our society rapidly ages, it is projected that by 2050, more than 82 million people in the United States will be older than 65 years of age (Bureau USC, 2023). Consequently, the prevalence of age-related diseases, including cardiovascular disease, type II diabetes, arthritis, neurodegenerative conditions such as Alzheimer’s disease and related dementias (ADRD), and cancer, is expected to rise (GBDPsD, 2018; Hawker & King, 2022; Joynt Maddox et al., 2024; Li et al., 2024; Zhang et al., 2021). The factors contributing to age-associated health decline are multifactorial, encompassing genetics, physical activity, socioeconomic status, and environmental and lifestyle influences such as alcohol consumption (Metti et al., 2018; Ortol et al., 2024; Schmitz et al., 2025; Zhao et al., 2023). Both clinical and experimental evidence demonstrate that non-resolving inflammation is implicated in many chronic diseases and is a central feature of aging (Chavda et al., 2024; Franceschi & Campisi, 2014; Furman et al., 2019). This chronic, low-grade inflammatory state, termed “inflamm-aging,” reflects heightened basal inflammation characteristic of older adults (Fulop et al., 2023). Notably, inflammation is a hallmark of both advanced age (Baechle et al., 2023) and alcohol misuse (Wang et al., 2010). Recent studies indicate that the combination of aging and alcohol use exacerbates immune dysfunction, promoting chronic hyperinflammatory states across multiple tissues (Anton et al., 2023, 2024; McMahan et al., 2021; Ren et al., 2022). Similarly, a cross-sectional study of Danish adults aged 50–64 found that heavy alcohol consumption was associated with nearly a twofold increase in the risk of hospitalization for pneumonia (Kornum et al., 2012).

Both advanced age and alcohol misuse are independently associated with intestinal barrier dysfunction and microbial dysbiosis. While it remains unclear whether these factors synergistically or additively drive the functional decline of the gut, several recent studies have highlighted their critical interaction in this process (McMahan et al., 2021, 2023). As previously discussed, a breach in intestinal barrier integrity promotes systemic inflammation, as microbiome-associated products that are normally confined to the intestinal lumen gain access to the circulation and interact with immune and non-immune cells across multiple tissues (Bishehsari et al., 2017; Di Vincenzo et al., 2024). Under normal circumstances, a competent immune system effectively contains and clears these translocated bacteria; however, impaired immune function allows for the expansion of bacteria beyond the mesentery, through the thoracic duct, and potentially into the systemic circulation, leading to bacteremia and systemic inflammation (Di Vincenzo et al., 2024). Experimental evidence demonstrates that aged mice exhibit increased susceptibility to ethanol-induced gut barrier dysfunction and inflammation, even at moderate levels of alcohol exposure, due, in part, to alterations in gut microbial composition and disrupted expression of antimicrobial genes (McMahan et al., 2023). Advanced age itself is an independent factor associated with reduced microbial diversity and shifts in microbial communities, including an enrichment of *Proteobacteria* (Enterobacteriaceae) and a decline in the relative abundance of beneficial bacteria such as *Actinobacteria* (*Bifidobacteria*) and *Firmicutes* (*Lactobacilli*), findings consistently reported in both clinical and preclinical studies (Claesson et al., 2012; Langille et al., 2014; Sommer et al., 2025; Xu et al., 2019). Multiple factors contribute to these age-related microbiome changes, including dietary modifications such as reduced fiber intake, poly-pharmacy, and antibiotic exposure. However, intrinsic biological changes within intestinal cells are likely central to this process (Funk et al., 2023; Walrath et al., 2021). For example, advanced age is associated with increased cellular senescence, characterized by irreversible cell cycle arrest and the accumulation of pro-inflammatory mediators collectively termed the senescence-associated secretory phenotype (SASP), including cytokines, chemokines, and proteases (Basisty et al., 2020; Franceschi & Campisi, 2014). Senescent intestinal epithelial and Paneth cells exhibit dysregulated production of antimicrobial peptides, diminishing the gut’s capacity to maintain microbial homeostasis (Forsyth et al., 2024; Jang et al., 2024). In addition, mucus production is dramatically impaired from senescent goblet cells leading to direct epithelial cell-microbiota interactions (Elderman et al., 2017; Sovran et al., 2019; Zheng et al., 2022). Together, increased SASP activity with reduced antimicrobial peptide and mucin production contribute to microbial dysbiosis and the chronic low-grade inflammation characteristic of “inflamm-aging.” Given that advanced age alone predisposes individuals to GI dysfunction and microbial dysbiosis, the added insult of alcohol misuse may represent a critical, modifiable risk factor for the acceleration of age-related disease onset.

The multiorgan effects of alcohol result from both direct and indirect mechanisms related to alcohol exposure. As previously discussed, the oxidative metabolism of ethanol produces reactive acetaldehyde and oxygen species, contributing to oxidative stress, lipid peroxidation, and DNA damage. Notably, these metabolic pathways are altered with advancing age, characterized by reduced expression and diminished capacity of enzymes responsible for ethanol metabolism (Meier & Seitz, 2008). This impaired ability to clear ethanol prolongs its presence in the blood and tissues, thereby increasing the duration of exposure and potential for damage (Anton et al., 2023; Seitz et al., 1989). However, altered ethanol clearance alone does not fully account for the heightened susceptibility to alcohol-related organ injury observed in older adults (Anton et al., 2024). Instead, age-associated biological changes, including chronic low-grade inflammation and increased cellular senescence, may more critically predispose older individuals to alcohol-induced harm, but further research is needed to support this claim.

Aging is associated with compromised integrity not only of the intestinal barrier but also of other epithelial barriers, including the blood-brain barrier (BBB), skin, respiratory tract, and renal epithelium (Parrish, 2017). This widespread epithelial vulnerability suggests that barrier dysfunction is a central mechanism contributing to age-related health decline. Supporting the hypothesis that the gut acts as a key coordinator of age- and alcohol-related pathology, increased circulating inflammatory and pathogen-associated mediators can further weaken the BBB (de Vries et al., 1996), enhancing its permeability during subsequent ethanol exposures (Vore & Deak, 2022; Wei et al., 2021). While studies have not directly examined the interaction between alcohol and advanced age at the BBB interface, evidence of increased neuroinflammation and neuronal injury following alcohol exposure points to BBB dysfunction as a critical pathobiological component of the gut-brain axis in the context of aging. Prolonged neuroinflammation and neuronal injury by alcohol have been implicated across several brain regions, including those involved in addiction, reward processing, craving, cognition, and memory (Leko et al., 2023; Lowe et al., 2020; Togre et al., 2024; Vetreno et al., 2021). However, there remains a significant gap in our understanding of how advanced age and alcohol together mechanistically influence addiction vulnerability and brain health across specific neural circuits. Despite these limitations, preclinical data increasingly support the concept that alcohol represents a modifiable risk factor for Alzheimer’s disease and related dementias (ADRD) in older populations (Anton et al., 2024, 2025a; Schwarzinger et al., 2018).

Collectively, given the significant gaps in knowledge in the field of alcohol and advanced aging research, it is understandable that therapeutic interventions remain in their early stages. Recognizing the central role of inflammation in this context, preclinical research has primarily focused on defining specific inflammatory pathways that could serve as feasible targets for future therapeutic strategies. One such pathway involves silent information regulator 1 (SIRT1), a NAD^+^-dependent deacetylase that plays a pivotal role in regulating aging and inflammation (Chen et al., 2023; de Gregorio et al., 2020; Jiao & Gong, 2020). SIRT1 is essential for maintaining several critical biological processes, including DNA repair, telomere maintenance, and metabolic homeostasis (Nogueiras et al., 2012). During aging, SIRT1 levels naturally decline, contributing to the accumulation of cellular damage and the progression of age-associated diseases (Mun, 2023). As a result, SIRT1 has emerged as a promising “anti-aging” target, with interventions such as resveratrol, NAD^+^ precursors, quercetin, and synthetic SIRT1 activators currently under investigation (Table 1) (Bonkowski & Sinclair, 2016; Sharma et al., 2023; Wicinski et al., 2023; Yang, Liu, et al., 2021). In the alcohol research field, the role of SIRT1 is well established (Ren et al., 2020); however, only a single study to date has provided strong evidence supporting the feasibility of SIRT1-targeted interventions in preclinical models examining the interaction of advanced age and alcohol exposure on immune function (Ren et al., 2022).

In addition to SIRT1, other inflammatory pathways under consideration include NF-κB and the NOD-like receptor protein 3 (NLRP3) inflammasome, both of which are implicated in driving “inflamm-aging” during advanced age and promoting chronic inflammation in models of ethanol exposure (de Carvalho Ribeiro et al., 2023; Li et al., 2022; Lippai et al., 2013; Sebastian-Valverde & Pasinetti, 2020; Zhong et al., 2016). Recent studies have explored therapeutic approaches aimed at suppressing both NF-κB and NLRP3 translation using antisense peptide nucleic acid-conjugated nanoparticles in preclinical models of binge ethanol exposure superimposed on advanced aging (Table 1) (Anton, Twardy, et al., 2025). These investigations demonstrate that such strategies can attenuate both age-related (Wahl et al., 2024) and alcohol-induced neuroinflammation (Anton, Twardy, et al., 2025). While these studies have confirmed the specificity of this approach in both the brain and peripheral tissues (Risen et al., 2024), the primary focus has remained on the brain, leaving important gaps in our understanding of how these interventions might impact peripheral components of “inflamm-aging” in these models.

Beyond targeting inflammation directly, restoring epithelial barrier integrity has emerged as another promising therapeutic strategy (Kong et al., 2024; Neurath et al., 2025; Toth et al., 2025). Potential interventions that have yet to be explored for alcohol- and age-related damage include approaches to correct microbial dysbiosis, enhance intestinal stem cell function, and promote tight junction assembly (Table 1) (Neurath et al., 2025). Additionally, as the accumulation of senescent cells, particularly dysfunctional intestinal epithelial and Paneth cells, contributes to impaired AMP production and barrier dysfunction, the use of senolytic therapies offers an exciting new direction (Table 1) (Gold et al., 2020; Luo et al., 2024; Saccon et al., 2021). As these agents selectively eliminate senescent cells, they may potentially restore epithelial barrier function, improve AMP production, and normalize gut dysbiosis associated with both advanced age and alcohol misuse. In conclusion, while therapeutic interventions remain at the frontier of research in alcohol and aging, emerging approaches targeting inflammation, epithelial integrity, and cellular senescence hold promise and warrant further investigation. Features such as intestinal barrier dysfunction, microbial dysbiosis (e.g., loss of beneficial taxa like *Bifidobacteria* and *Lactobacilli*), increased pro-inflammatory mediators associated with SASP, and alterations in pathways such as SIRT1, NF-κB, and NLRP3 may serve as candidate biomarkers. Furthermore, these may hold potential to be used in combination with non-invasive, organ-specific damage markers (e.g. osteopontin (Du et al., 2022), mesencephalic astrocyte-derived neurotrophic factor (Liu et al., 2022), c-reactive protein (Luan & Yao, 2018), neurofilament light chain (Pham et al., 2025), glial fibrillary acid protein (Abdelhak et al., 2022), brain-derived neurotrophic factor (Pham et al., 2025)) to inform both the diagnosis of alcohol-related harm as well as the prognostic potential of future interventions. These elements, though largely preclinical at present, offer promising avenues for the identification of early indicators of alcohol-related vulnerability in aging populations. Selectively targeting the GI tract may not only mitigate alcohol-related damage within the gut but also reduce secondary injury to other organs, including the liver, lung, and brain, offering a novel and multi-system strategy for improving health outcomes in older adults with alcohol misuse.

## 2. Conclusion

**Fig. 1.**
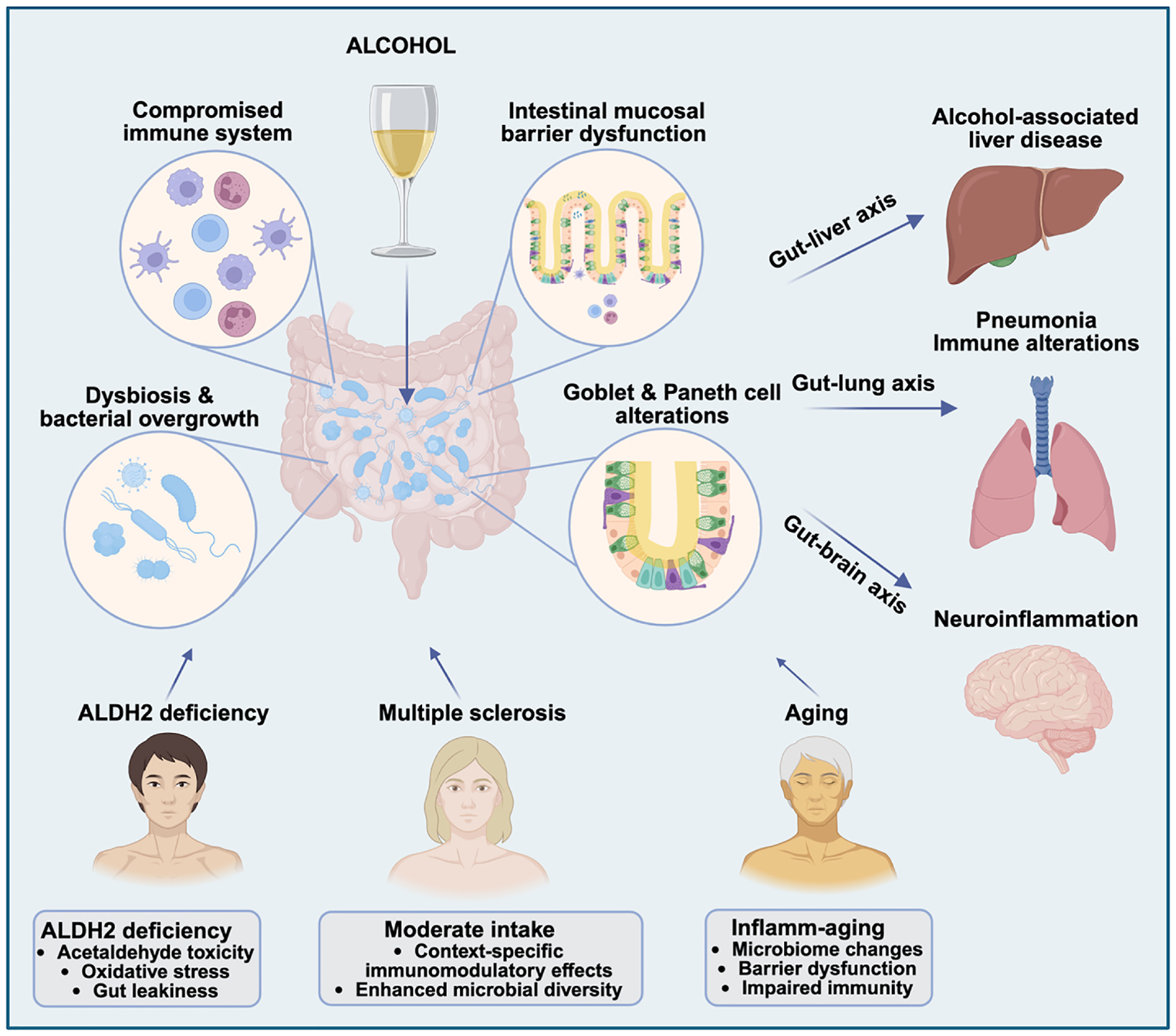
Alcohol effects on host physiology and disease through the gut–liver, gut–lung, and gut-brain axes. Chronic and excessive alcohol intake disrupts intestinal immune homeostasis, alters gut microbial ecology, impairs epithelial cell function—including goblet and Paneth cells—and compromises mucosal barrier integrity. These changes promote microbial dysbiosis, bacterial overgrowth, and translocation of microbial products, thereby fueling systemic inflammation through the gut–liver, gut–lung, and gut–brain axes. In the gut–lung axis, such alterations result in pulmonary immune cell infiltration, oxidative stress, and an increased susceptibility to respiratory infections (e.g., pneumonia), whereas in the gut–liver axis they promote enhanced inflammation and the progression of ALD. In this context, aldehyde dehydrogenase 2 (ALDH2) deficiency exacerbates ethanol hepatotoxicity by amplifying acetaldehyde accumulation, oxidative stress, and intestinal permeability. Conversely, moderate alcohol consumption may exert context-specific immunomodulatory effects, attenuating inflammation and enhancing microbial diversity in autoimmune diseases, such as multiple sclerosis, thereby demonstrating a dose-dependent effect on host physiology. However, potential interactions with immunomodulatory medications and the risk of synergistic toxicity must be carefully considered. Aging further amplifies alcohol’s detrimental effects by promoting chronic low-grade inflammation (“inflamm-aging”), epithelial senescence, impaired antimicrobial defenses, and a decline in microbial diversity—all of which increase susceptibility to alcohol-associated disease. Precision-based interventions targeting the gut microbiome, epithelial barrier integrity, cellular senescence, and genotype-specific vulnerabilities may offer promising therapeutic strategies. Elucidating the mechanistic underpinnings of the alcohol–microbiome–immune axis across the lifespan is essential to guide interventions that reduce harm while harnessing the potential therapeutic effects of microbiome modulation in select clinical contexts. Figure created with a BioRender license.

Alcohol consumption influences human health through complex interactions involving genetic factors, immune regulation, the gut microbiome, and aging mechanisms (Fig. 1). Genetic polymorphisms, such as ALDH2 deficiency, impair acetaldehyde detoxification and promote oxidative stress, intestinal barrier dysfunction, and microbiota dysbiosis, even at low alcohol exposure. Chronic or heavy alcohol use amplifies these alcohol-related effects, leading to systemic inflammation and increased risk of organ damage, such as liver, lung, and brain injury. Aging further exacerbates these vulnerabilities by driving chronic low-grade inflammation, epithelial senescence, and impaired mucosal immunity, resulting in increased intestinal permeability and altered microbial composition. Dysbiosis, intestinal barrier dysfunction, microbial translocation, endotoxemia, and immune dysregulation contribute to disease pathogenesis in the gut–liver, gut–lung, and gut–brain axes.

Emerging evidence suggests that moderate alcohol consumption may confer immunomodulatory effects in certain autoimmune diseases. Current observational studies must be validated with well-designed prospective human trials and mechanistic animal studies to distinguish between different alcoholic beverages, alcohol dosing, and account for confounding lifestyle factors in adults across the lifespan. Such research could unlock a promising therapeutic frontier in autoimmune diseases by enabling the development of targeted interventions that replicate alcohol’s beneficial immunomodulatory effects without the inherent risks of alcohol consumption.

The complex, bidirectional relationship between alcohol use, the gut microbiome, immune regulation, and aging underscores the need for an integrated, systems-level understanding of alcohol-related disease. Across diverse contexts, from ALD and neuroinflammation to autoimmune conditions and age-related pathologies, the gut and its microbial communities play a key role in ALD, lung, and neurological diseases. Future research must continue to dissect the nuanced mechanisms underlying these interactions, with a focus on developing precision-based, gut-targeted interventions that not only mitigate alcohol-related harm but also harness the therapeutic potential of microbiome modulation. Such approaches hold promise for improving health outcomes in both younger and older adults across the spectrum of alcohol exposure.

## Figures and Tables

**Table 1:** Summary of microbiome- and inflammation-targeted interventions.

Intervention	Mechanism/Target	Supporting Model or Clinical Data
**Fecal Microbiota Transplantation (FMT)**	Restores microbial diversity, reverses dysbiosis	Encouraging outcomes in patients with severe alcohol-associated hepatitis; lesser ascites, infections, encephalopathy, and alcohol relapse (with a trend toward higher survival rates) (Philips et al., 2022).
**2’-Fucosyllactose (fucosylated oligosaccharide)**	Enhances α1–2-fucosylation of intestinal mucins; suppresses *E. faecalis* overgrowth and cytolysin production	Fut2-deficient mice show increased ALD severity; 2’-fucosyllactose reduces liver damage and microbiome dysbiosis in preclinical alcohol models (Zhou et al., 2020)
**Bacteriophage Therapy**	Targeted depletion of cytolysin positive *Enterococcus faecalis* pathobiont	Cytolysin positivity predict mortality in patients with alcohol-associated hepatitis; bacteriophage therapy against cytolysin positive *Enterococcus faecalis* prevents ethanol-induced liver disease in preclinical models (Duan et al., 2019).
**SIRT1 Activation**	Enhances DNA repair, telomere maintenance, reduces inflammation, regulates metabolic homeostasis (Nogueiras et al., 2012)	Resveratrol, NAD^+^ precursors, quercetin, and synthetic activators currently under investigation in preclinical models (Bonkowski & Sinclair, 2016; Sharma et al., 2023; Wicinski et al., 2023; Yang, Liu, et al., 2021).
**NF-**κ**B and NLRP3 Inflammasome Inhibition**	Suppresses pro-inflammatory gene transcription and blocks IL-1β/IL-18 activation, reduces systemic inflammation and neuroinflammation (de Carvalho Ribeiro et al., 2023; Li et al., 2022; Lippai et al., 2013; Sebastian-Valverde & Pasinetti, 2020; Zhong et al., 2016).	Inhibition with antisense peptide nucleic acid-conjugated nanoparticles reduced ethanol- and aging-induced neuroinflammation in mice (Anton, Twardy, et al., 2025; Wahl et al., 2024).
**Tight Junction Enhancers**	Improve intestinal barrier function, prevent microbial translocation (Zhou & Zhong, 2017).	Investigational; efficacy shown in alcohol (Cresci et al., 2017; Zhong et al., 2015) and aging models (Kuhn et al., 2020; Neurath et al., 2025)
**Senolytic Therapy**	Eliminates senescent epithelial and Paneth cells to restore AMP production and epithelial barrier integrity; reduces dysbiosis (Eskiocak et al., 2024; Jang et al., 2024)	Mouse studies show improved mucosal immunity, gut barrier function, and reduced inflammation in aging or alcohol contexts (Gold et al., 2020; Luo et al., 2024; Saccon et al., 2021)

## Abbreviations:

ADH: Alcohol dehydrogenase
AUD: alcohol use disorder
ALD: alcohol-associated liver disease
ABD: alcohol-related brain damage
ALDH2: aldehyde dehydrogenase 2
ADRD: Alzheimer’s disease and related dementias
AMPs: antimicrobial peptides
BBB: blood-brain barrier
CYP2E1: cytochrome P450–2E1
DCs: dendritic cells
EAE: experimental autoimmune encephalomyelitis
FMT: fecal microbiota transplantation
GI: gastrointestinal
GAPs: goblet cell-associated antigen passages
ILC3: group 3 innate lymphoid cells
IL: interleukin
KO: knockout
MEOS: microsomal ethanol-oxidizing system
MUC2: Mucin-2
mAChR4: muscarinic ACh receptor 4
NADPH: nicotinamide adenine dinucleotide phosphate
NAD^+^: nicotinamide adenine dinucleotide
NK: natural killer
NLRP3: NOD-like receptor protein 3
NF-κB: nuclear factor kappa B
PAMP: pathogen-associated molecular pattern
PUFAs: polyunsaturated fatty acids
ROS: reactive oxygen species
Reg3: regenerating antimicrobial peptides
RA: rheumatoid arthritis
SASP: senescence-associated secretory phenotype
SCFAs: short-chain fatty acids
SIRT1: silent information regulator 1
TNF-α: tumor necrosis factor-alpha
WT: wild-type
